# Unraveling the mediation role of frailty and depression in the relationship between social support and self-management among Chinese elderly COPD patients: a cross-sectional study

**DOI:** 10.1186/s12890-024-02889-y

**Published:** 2024-02-01

**Authors:** Jie Zhao, Xiaona Zhang, Xindan Li, Rui Zhang, Yan Chang, Yongju Li, Hongyan Lu

**Affiliations:** 1 Department of Master’s Training Station, General Hospital of Ningxia Medical University, Yinchuan, 750004 China; 2 Department of Nursing, General Hospital of Ningxia Medical University, Yinchuan, 750004 China

**Keywords:** COPD, Social support, Frailty, Self-management, Depression, Mediation effect

## Abstract

**Background:**

Self-management (SM) is the key factor in controlling the progression of chronic obstructive pulmonary disease (COPD). Previous studies have reported that majority of COPD patients later presented with frailty and mental health diseases, which affect self-management. This study attempted to explore the mediation role of depression and frailty between social support and self-management in elderly COPD population.

**Methods:**

Six hundred twenty-seven stable elderly COPD patients admitted to 5 public hospitals in Ningxia, China were selected as study subjects by convenience sampling method. Self-management, frailty, depression and social support were assessed using the COPD Self-management Scale (COPD-SMS), Frail Scale (FS), 15-item Geriatric Depression Scale (GDS-15), and Social Support Rating Scale (SSRS) respectively. The Pearson correlation analysis was used to assess the correlation between variables. Additionally, SPSS25.0 PROCESS plugin Model 6 was used to explore the mediating effects of frailty and depression in the relationship between social support and self-management.

**Results:**

The mean participant age was 72.87 ± 7.03 years, 60.4% of participants were male. The mean total score of the COPD-SMS was 156.99 ± 25.15. Scores for the SSRS, FS, and GDS-15 were significantly correlated with COPD-SMS (*p* < 0.05). The analysis of the mediation effect demonstrated that social support has a direct predictive effect on self- management (β = 1.687, 95%CI: 1.359 to 2.318). Additionally, social support can also predict self- management indirectly through the mediation of depression (β = 0.290, 95%CI: 0.161 to 0.436) and frailty-depression (β = 0.040, 95%CI: 0.010 to 0.081). However, the mediation effect of frailty alone was not found to be statistically significant (β =—0.010, 95%CI: -0.061 to 0.036). The direct effect accounted for 84.06% of the total effect, while the indirect effect accounted for 15.94% of the total effect.

**Conclusion:**

Self-management among elderly COPD patients was relatively moderate to low. Furthermore, frailty and depression were found to have a partially mediation role in the relationship between social support and self-management. Therefore, healthcare professionals need to comprehensively consider the frailty and depression status of patients, and implement targeted intervention measures as part of their care, which can improve the self-management of elderly COPD patients.

## Background

Chronic obstructive pulmonary disease (COPD) is a condition characterized by persistent airway inflammation and airflow limitation [1]. Approximately 20% of the global adult population suffers from COPD, with over 80% of related deaths occurring in low- and middle-income countries or regions such as China, India, and Southeast Asia [2–4]. Currently, no effective cure way for COPD has been found, and its treatment primarily focuses on stabilizing the condition and alleviating symptoms [1]. Self-management (SM) is a key factor in enhancing the quality of life and health outcomes for COPD patients, referring to the individual’s abilities to manage symptoms, treatment, physical and psychosocial consequences, and lifestyle changes inherent in living with a chronic disease [5]. The 2023 Global Initiative for Chronic Obstructive Lung Disease guidelines [1] and Cochrane Review [6] have both affirmed the benefits of self-management in improving indicators such as PaO_2_, PaCO_2_, and lung capacity in COPD patients. However, previous studies have focused on exploring multiple influencing factors of self-management, and there is insufficient evidence regarding the intrinsic mechanisms between self-management and its influencing factors [7, 8]. According to Bandura’s social cognitive theory, behavioral change is the result of the interaction between external environmental factors and internal characteristics [9]. Social support is an important external factor for behavior change. It encompasses the perceived availability of social resources from individuals' social network members, and empowers patients, boosts their confidence in facing illness, promotes healthy behaviors, and directly or indirectly predicts self-management [10, 11].

Frailty is a group of syndromes caused by aging and various chronic diseases that leads to degenerative changes, increases vulnerability of the body, affects patients' physical, psychological, and social functions [12]. Elderly COPD patients are prone to experience dyspnea after activities, leading to a long-term reduction in physical activity, which results in decreased muscle mass and function. The incidence of frailty is as high as 25.60% to 44.70% [13–15]. Some evidence has shown that patients with high levels of social support are less likely to experience frailty [16]. What’s more, there is a correlation between frailty and self-management [17]. Therefore, as an individual factor, frailty may play a mediation role in the pathway of social support and self-management.

In addition to frailty, depression is another common complication of people diagnosed with COPD. COPD patients who use medications such as theophylline and steroids for a long period of time, as well as those with low arterial oxygen saturation leading to periventricular white matter lesions, are more prone to depression [1]. According to reports, 80% of elderly COPD patients suffer from depression, and COPD patients with frailty experience even more severe depression [18, 19]. Many studies have shown that an improved social support system can alleviate patients’ psychological stress reactions, enhance proactive disease management, and predict self- management [20]. Therefore, depression may serve as another mediation variable between social support and self-management. Unfortunately, most of the research findings were about effect of social support and self-management on depression, frailty, etc. [6, 8], while lacking exploration of the potential underlying mechanisms relating depression, frailty, and their connection to social support and self- management.

**Fig.1:**
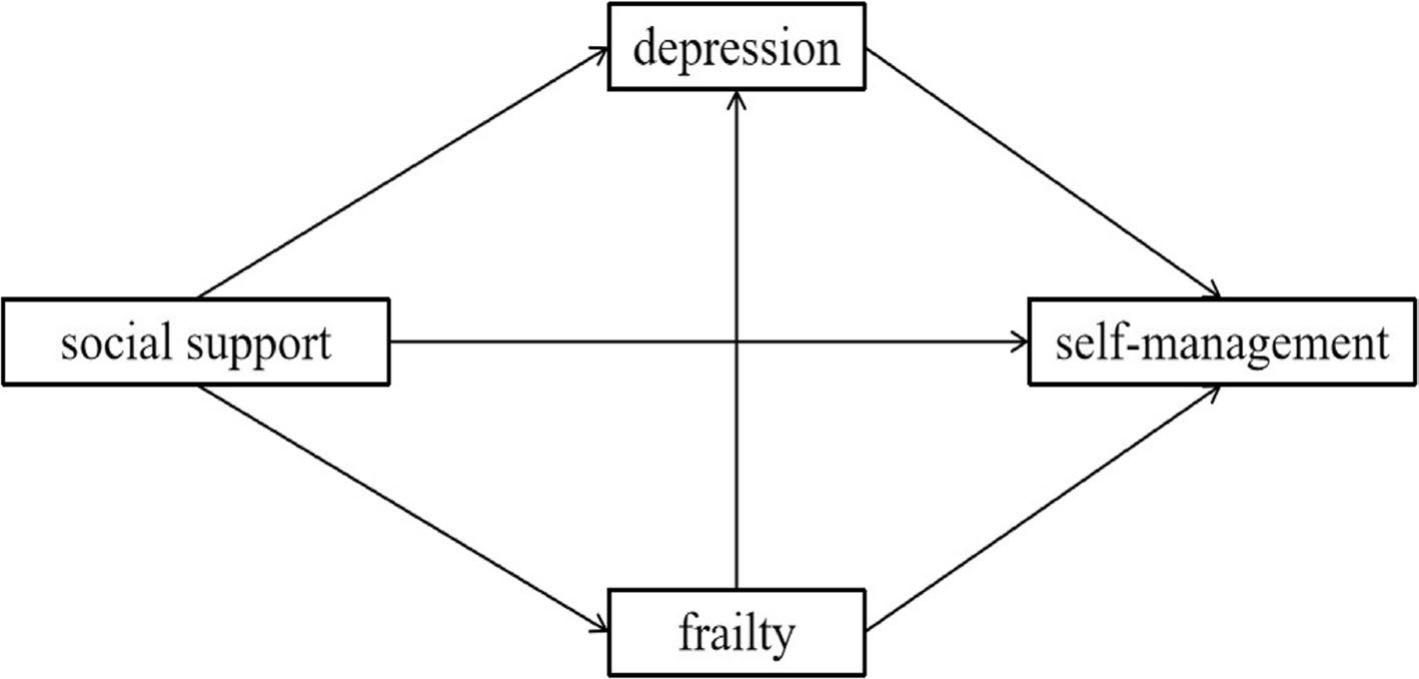
Research framework of the relationship between social support and self-management

Therefore, we will develop and validate a hypothetical model (Fig. 1) to explore the underlying mechanisms and the strength of the effects of frailty and depression on the social support and self-management pathways in elderly COPD patients. It is expected that based on the findings, researchers can formulate more targeted care plans and interventions to enhance the biopsychosocial health of elderly COPD patients, and improve their self-management abilities.

## Methods

### Study design and participants

In this cross-sectional study, a convenience sampling was used to surrey elderly COPD patients as the research subjects who were in a stable phase. The patients were selected from 5 public hospitals in the Ningxia region of northwest China during November 2020 to July 2021. The inclusion criteria for this study were as follows: (1) Who met the diagnostic criteria for COPD according to the guidelines of the Global Initiative for Chronic Obstructive Lung Disease [1] and were in a stable period; (2) Individuals aged 60 years or older, diagnosed with COPD for at least 6 months; (3) All subjects were fully informed about the study and provided signed informed consent. The exclusion criteria were as follows: (1)Those combined severe cardiovascular and cerebrovascular diseases, severe liver and kidney diseases, malignant tumors (such as lung cancer), and other end-stage diseases; (2)Those had cognitive impairment, unclear consciousness, or a history of mental illness.

This study was a cross-sectional survey, the sample size based on the reference of 10 times the items [21]: the questionnaire included 48 items, and considered invalid questionnaires, the sample size was expanded by 20%, and the sample size was determined to be 576 cases.

### Measures

This study utilized literature research, expert group meetings, and other methods to develop a self-designed questionnaire for elderly COPD patients, which included: (1) General information questionnaire: It was designed by the researcher in accordance with the research purpose and content. This section included demographic data such as gender, age, body mass index (BMI), current residence, and marital status; Disease-related information: This section covered aspects related to the disease, including the disease duration and the number of acute exacerbations; (2) COPD-SMS: This scale was developed by Chinese scholar Zhang [22] and included 5 dimensions: symptom management, information management, daily life management, emotional management, and self-efficacy. With scoring options on a Likert scale (0 to 5) and a total possible sum of 51 to 255 (where higher scores indicated better the patients' self-management status). Cronbach's α is 0.92; (3) FS: This scale was developed by experts from the International Association for Nutrition, Health, and Aging [23]. This study used a version of the scale adapted to the Chinese population by Wei [24]. The questionnaire consisted of 5 items (fatigue, decreased endurance, limited mobility, comorbidity, and decreased body mass) rated on a scale of 0 and 1. Total scores range from 0 to 5, with higher scores indicated a higher risk of frailty. The Cronbach's α is 0.826; (4) GDS-15: This scale is a simplified version of the geriatric depression scale developed by Sheikh [25], used to quickly assess the depression status of elderly people. This study adopted the Chinese version revised by Tang [26]. The higher the score, the higher the risk of geriatric depression. GDS-15 has been translated into various languages and shown to have satisfactory validity and reliability. The Cronbach's α is 0.793; (5) SSRS: This scale was developed by Xiao [27], and and it includes 3 dimensions with a total of 10 items. The scale utilizes a 4-level and multiple scoring method, where single choice questions are scored based on the number of selected items, and multiple choice questions are scored based on the number of selected items. The total score ranges from 12 to 66 points. A higher score indicates a higher level of social support. The Cronbach’s α coefficient for this scale is 0.91.

Before the survey begins, the researcher obtained permission and cooperation from the responsible person of the target hospital and department. The researcher distributed questionnaires on-site to conduct the survey, obtained consent from the research subjects, and had them sign a written informed consent. After the survey was completed, the researcher checked the questionnaires on-site for any missing items. After verifying that there were no errors, the questionnaires were saved. Two researchers coded and inputted the data for statistical analysis, and a third person conducted spot checks on all the data. For individual missing values in the data, the mean of the variable would be used as a replacement.

### Statistical analysis

All statistical analyses were performed using IBM SPSS Statistic v26.0 software (SPSS Inc., Chicago, IL, USA). The following approaches were used for data presentation and analysis: (1) Continuous variables with normal distribution were presented as the means and standard deviation (M ± SD), and the count data were expressed by the number of cases and percentage. (2) The Pearson correlation analysis was used to assess the correlation between variables. (3) Mediating effect was statistically analyzed using PROCESS Model 6 developed by Hayes [28], and bootstrapping 5000 resamples with a 95% CI was used to analyze the significance of mediating model. Consistent with Hayes’ recommendations, we determined the statistical significance of the mediation effects by examining the 95% CI, which excluded 0. The *P* ≤ 0.05 was considered as statistically significant.

## Results

### Descriptive analysis

A total of 627 elderly COPD patients were invited, 379 males and 248 females were included in the analysis. Mean (SD) age of the participants was 72.87(± 7.03) years; most lived in a rural area (63.64%). The majority of participants had not received education beyond elementary school (46.57%). Additionally, only 29.51% of participants had a per capita monthly income exceeding 3000 RMB, and 69.38% of the patients used inhalers for treatment (Table 1).

**Table 1:** Descriptive statistics of participants and study variables (*N* = 627)

Variable	Category	N(%)
Age(year)	60–69	220(35.09)
	70–79	280(44.66)
	≥ 80	127(20.25)
Gender	Male	379(60.45)
	Female	248(39.55)
BMI group	< 18.5	46(7.34)
	18.5–23.9	309(49.28)
	24.0–27.9	200(31.90)
	≥ 28.0	72(11.48)
Residence	Urban	228(36.36)
	Rural	399(63.64)
Primary caregiver	Spouse	281(44.82)
	Children or others	179(28.55)
	Self-care	167(26.63)
Education (school)	≥ College	20(3.19)
	High	52(8.29)
	Middle	111(17.70)
	Elementary	152(24.24)
	< Elementary	292(46.57)
Per capita monthly income(RMB)	≤ 1000	151(24.08)
	1001 ~ 2000	192(30.62)
	2001 ~ 3000	99(15.79)
	> 3000	185(29.51)
Type of health insurance	Self-funded	14(2.23)
	Medical insurance for urban and rural residents	433(69.06)
Variable	Category	N(%)
	Medical insurance for urban employees	180(28.71)
Course of disease (year)	< 5	281(44.82)
	5 ~	167(26.63)
	> 10	179(28.55)
Acute exacerbation in the past year (times)	< 2	449(71.61)
	≥ 2	178(28.39)
Smoking	Never	366(58.37)
	Former	239(38.12)
	Daily	22(3.51)
Inhalant	Yes	435(69.38)
	No	192(30.62)
Exercise (minutes/time)	≤ 30	470(74.96)
	> 30	157(25.04)
Exercise (times/week)	< 3	383(61.08)
	≥ 3	244(38.92)
Respiratory muscle training	Yes	445(70.97)
	No	182(29.03)

### Exploring the correlation between social support, self-management, frailty, and depression

As shown in Table 2, the correlation among social support, self-management, frailty, and depression in elderly patients with COPD in this study revealed the following mean values: SSRS (mean = 35.47, SD = 5.66), COPD-SMS (mean = 156.99, SD = 25.15), FS (mean = 2.59, SD = 1.09), and GDS-15 (mean = 6.58, SD = 2.67). The correlation analysis indicated significant associations between social support (*r* = 0.451, *p* < 0.01), frailty (*r* = -0.109, *p* < 0.01), and depression (*r* = -0.334, *p* < 0.01) with self-management in patients with COPD. These findings serve as a foundation for constructing structural equation models.

**Table 2:** Mean, Standard Deviation, and Correlations among the social support, frailty, depression and self-management in patients with COPD (*N* = 627)

Variable	1	2	3	4	M ± SD
1.social support	1				35.47 ± 5.66
2.frailty	-0.118^**^	1			2.59 ± 1.09
3.depression	-0.363^**^	0.409^**^	1		6.58 ± 2.67
4.self-management	0.451^**^	-0.109^**^	-0.334^**^	1	156.99 ± 25.15

*M* mean, *SD* standard deviation

^**^p < 0 .01

### The mediating effect of frailty and depression between social support and self-management in elderly COPD patients

This study has taken social support as the independent variable, self-management of elderly COPD patients as the dependent variable, and frailty and depression as mediating variables.

**Fig. 2:**
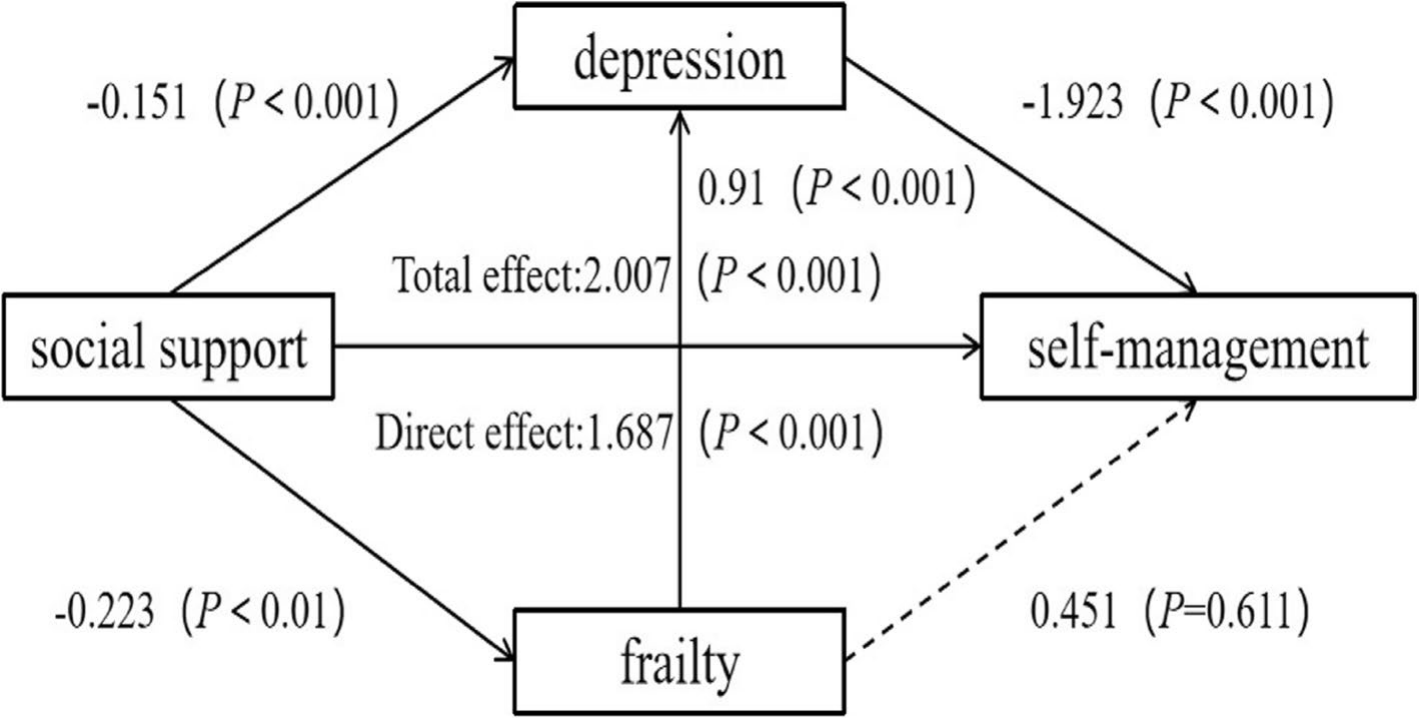
The mediating role of frailty and depression in the relationship between social support and self-management among elderly COPD patients

In the prediction of self-management behavior in elderly COPD patients with social support, two pathways were identified as mediating effects. Figure 2 and Table 3 showed that the first pathway, involving depression (indirect effect 2), and the second pathway, involving frailty-depression (indirect effect 3), were found to be statistically significant. However, the independent mediating effect of frailty (indirect effect 1) was not significant.

**Table 3:** Understanding the influence of depression and frailty on social support and self-management in elderly patients with COPD (*N* = 627)

Outcome variables	Predictor variables	R	R^2^	F	β	t
Self-management		0.451	0.204	159.705***	2.007	12.637***
	social support					
Frailty		0.118	0.014	8.823**		
	social support				-0.023	-2.970**
Depression		0.518	0.268	114.324***		
	social support				-0.151	-9.258***
	frailty				0.91	10.779***
Self-management		0.487	0.237	64.556***		
	social support				1.687	10.093***
	frailty				0.451	0.509
	depression				-1.923	-4.99***

^**^p < 0 .01

^***^p < 0 .001

### Bootstrapping results of the mediation model between social support and self-management in elderly COPD patients with frailty and depression

Table 4 showed that indirect effect 2 (β = 0.290, 95%CI: 0.161 to 0.436) and indirect effect 3 (β = 0.040, 95%CI: 0.010 to 0.081); but unlike the original hypothesis, the indirect effect 1 (β =—0.010, 95%CI: -0.061 to 0.036) is not significant as the 95% CI includes 0. The total indirect effect, which combines the effects of depression and the frailty- depression pathway, accounts for 15.94% of the total effect. Conversely, the direct effect of social support alone accounts for 84.06% of the total effect.

**Table 4:** Analysis of the mediating effect of frailty and depression on social support and self-management in elderly patients with COPD (*N* = 627)

Effect relation	Path	Estimated value	LLCI	ULCI	Relative effect size
Indirect effect 1	social support → frailty → SM	-0.010	-0.061	0.036	-
Indirect effect 2	social support → depression → SM	0.290	0.161	0.436	14.45%
Indirect effect 3	social support → frailty → depression → SM	0.040	0.010	0.081	1.99%
Total indirect effect		0.320	0.182	0.476	15.94%
Direct effect	social support → SM	1.687	1.359	2.015	84.06%
Total effect		2.007	1.695	2.318	

*LLCI* lower level for confidence interval, *ULCI* upper level for confidence interval

## Discussion

This study included a large sample of elderly patients with COPD in the Ningxia region of northwest China to understand the current state of SM, and explore the mediation role of depression and frailty between social support and SM in elderly patients with COPD.

First, this study uncovers that the self-management ability of elderly COPD patients in the Ningxia region is at a moderate-to-low level, which aligns with the research findings of Huang [29]. This condition can be attributed to a significant proportion of patients living in rural areas. Limited access to high-quality medical resources after discharge, due to constraints imposed by the medical environment and economic conditions, may have hindered patients from fully utilizing them. In addition, the low educational level of the patients makes it challenging for them to navigate and screen useful information regarding disease management from complex network information and specialized medical knowledge. This may lead to the development of misconceptions among the patients [30]. Furthermore, the current chronic disease self-management projects in China lack systematic and standardized evaluation methods and indicators. As a result, medical personnel are unable to systematically and comprehensively assess patients [31], leading to a lack of individualized and targeted implementation of SM interventions. Therefore, it is crucial for medical personnel to actively promote discharge planning and provide patients with disease treatment and self-management knowledge through various channels, including internet platforms. By expanding the avenues through which patients can access beneficial health information, it becomes possible to ensure that they receive accurate and understandable information regarding their recuperation. Second, the results of this study indicate that the social support of elderly COPD patients in the Ningxia region is at a moderate level. Social support, depression, and SM are correlated, which is consistent with the research findings of Korpershoek [32] and Hernández [33]. Furthermore, depression acts as a mediating variable between social support and self-management use, indicating that individuals with good social support and without depression are more likely to engage in higher levels of self-management activity. Patients with high social support may actively seek help, form partnerships with healthcare professionals, enhance their self-efficacy in coping with diseases, alleviate negative emotional distress, and ultimately transform unhealthy behaviors [34]. It is also worth noting that frequent acute exacerbation leads to rapid decline in lung function, lifestyle changes, and an increased incidence of depression in patients [35, 36]. According to the self-efficacy theory, patients suffering from negative emotions will lose confidence in their ability to manage diseases, and self-efficacy will decrease, which will affect self-management ability [37]. Sohanpal [38] pointed out that focusing on individual cognition, behavior, and symptoms related to depression can improve the SM level of COPD patients. Numerous pieces of evidence have been confirmed that physical and mental exercise can alleviate depression in COPD patients, improve their lung function and exercise ability [39, 40]. In summary, medical personnel need to promptly assess the depression status of elderly COPD patients, pay attention to personal cognition, behavior, and symptoms may relate to depression, and strengthen the education and supervision of physical and mental exercise for patients based on existing evidence.

Third, the findings suggest that frailty in elderly COPD patients is correlate with social support and self-management, consistent with the studies’ results of Hernández [33] and To [41]. However, frailty cannot be used as an independent variable to predict patient self-management, but can only played a chain mediating effect through depression enhancement. Previous studies have been demonstrated that the transition from a healthy state to a frailty state is a dynamic process, and frail elderly COPD patients are usually in a state of reduced physiological reserves, prone to anxiety and/or depression, exacerbate the occurrence of adverse health outcomes and affect patient health behavior [42, 43]. Although the progression of COPD damages multiple organ systems, leading to symptoms such as muscle weakness and osteoporosis, and increasing the risk of dynamic progression of frailty, these characteristics can also serve as targets for intervention. Maddocks [15] stated that COPD patients significantly improved their frailty state during lung rehabilitation treatment. In addition, exercise and nutritional interventions have a significant impact on improving frailty [44]. These findings suggest that medical personnel need to early identify the patients' frailty state and provide targeted interventions, combined with its dynamic changes, to detect the trend from health to frailty as soon as possible, block the path of depression triggered by frailty affecting self-management.

There were some noteworthy limitations to this study. First, the design of the study was a cross-sectional survey, and some items in the questionnaire had recall bias. Second, this study only selected many environmental and individual factors for mediation research, but the mechanisms of self-management behavior change are complex. In the future, longitudinal research is needed to explore the self-management level, influencing factors, and their change trajectory of elderly COPD patients, and to study the internal mechanisms that affect the patients’ self-management from different perspectives.

## Conclusion

To sum up, we found that the self-management of elderly COPD patients in the Ningxia region of northwest China was at a lower than average level. Depression and frailty-depression had a significant mediating effect between social support and self-management, but the independent mediating effect of frailty was not significant. The findings reminded that medical personnel need provide more targeted support environments for patients during implementation, and strengthen the identification, professional care, and health guidance of patients with frailty and/or depression to improve their self-management abilities.

## Data Availability

The data that support this study are available from the corresponding author upon reasonable request.

## Abbreviations

BMI: Body mass index
CI: Confidence interval
COPD: Chronic obstructive pulmonary disease
COPD-SMS: COPD self-management scale
FS: Frail Scale
GDS-15: 15-Item geriatric depression scale
SD: Standard Deviation
SM: Self-management
SSRS: Social support rating scale
